# Recent advancements of covalent organic frameworks (COFs) as proton conductors under anhydrous conditions for fuel cell applications

**DOI:** 10.1039/d3ra04855a

**Published:** 2023-10-16

**Authors:** Vellaichamy Joseph, Atsushi Nagai

**Affiliations:** a Ensemble3 – Centre of Excellence Wólczyńska 133 01-919 Warszawa Poland atsushi.nagai@ensemble3.eu

## Abstract

Recent electrochemical energy conversion devices require more advanced proton conductors for their broad applications, especially, proton exchange membrane fuel cell (PEMFC) construction. Covalent organic frameworks (COFs) are an emerging class of organic porous crystalline materials that are composed of organic linkers and connected by strong covalent bonds. The unique characteristics including well-ordered and tailorable pore channels, permanent porosity, high degree of crystallinity, excellent chemical and thermal stability, enable COFs to be the potential proton conductors in fuel cell devices. Generally, proton conduction of COFs is dependent on the amount of water (extent of humidity). So, the constructed fuel cells accompanied complex water management system which requires large radiators and airflow for their operation at around 80 °C to avoid overheating and efficiency roll-off. To overcome such limitations, heavy-duty fuel cells require robust proton exchange membranes with stable proton conduction at elevated temperatures. Thus, proton conducting COFs under anhydrous conditions are in high demand. This review summarizes the recent progress in emerging COFs that exhibit proton conduction under anhydrous conditions, which may be prospective candidates for solid electrolytes in fuel cells.

## Abstract 2

Characteristics of COFs and their proton conducting mechanisms.

## Graphical abstract

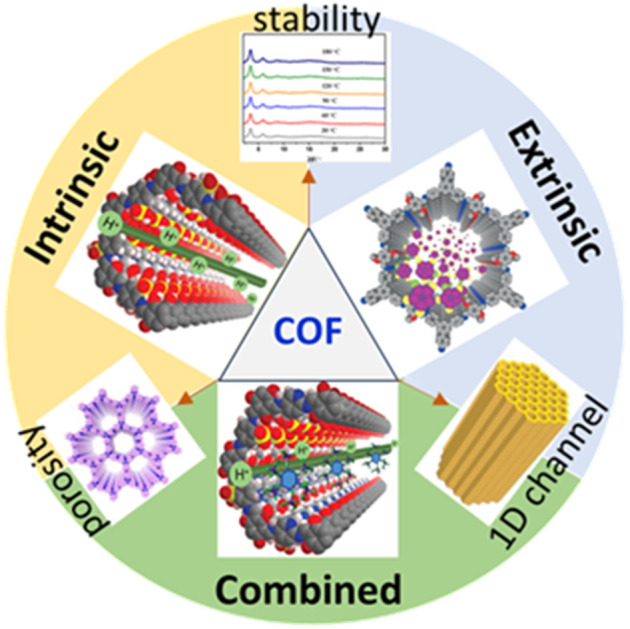

## Introduction

In the recent decades, development of renewable energy technologies aims to reduce the dependence on conventional fossil fuels for global energy demands, provide an alternative and sustainable way to meet the energy crisis. Among them, fuel cell technology grabs immense attention in the recent years owing to their unique characteristics such as high energy conversion efficiency, very low to zero hazardous carbon emission, fuel flexibility and long durability.^1,2^ Fuel cell is a device that directly converts chemical energy into electrical energy while leaving no hazardous by-products but only water. Typically, a prototype fuel cell consists of electrolytes sandwiched between two electrodes namely anode and cathode. Fuel cell produces electrical energy by means of consumption of fuel (*e.g.*, hydrogen) and oxygen, leaving water and heat as by-products. In principle, the generated electrons flow through circuit externally reaching cathode by producing electricity (*i.e.*, flow of electrons). On the other hand, generated protons have to reach cathode by passing through the electrolyte (proton conducting material). Several classes of materials such as metal–organic frameworks,^3^ covalent organic frameworks,^4^ conjugated microporous polymers,^5,6^*etc.*, were demonstrated as proton conducting materials. Among them, covalent organic frameworks (COF) are considered as potential candidates for their unique characteristics including but not limited to light weight elements attached through covalent bonding, high proton conduction along 1D nano channels, wide variety of customizable building blocks to tune structural and functional features.^7–9^ COFs are 2D or 3D crystalline materials tailored by lighter elements such as boron, carbon, nitrogen and oxygen, through covalent bonds in a periodic manner.^10,11^ Further, they exhibit high thermal stability, membrane processability, and excellent stability in harsh environments like strong acidic/basic conditions. Generally, COFs have high synthetic flexibility and structural diversity over organic porous materials and possess high stability over metal–organic frameworks. COFs generate unidirectional porous channels that provide high surface area and low density. COF skeleton is regulated by the linker size, and its symmetry. Further, linker symmetry determines the pore size, pore volume and their stacking pattern. They are counterparts of metal–organic frameworks (MOFs) in which transition metals attached to organic groups through coordination bond.^12,13^ Recent successes witnessed COF's flourishing potential for applications in diverse fields such as gas adsorption and separation,^14^ catalysis,^15,16^ sensors,^17^ electrochemical energy conversion and storage,^18,19^ and optoelectronics.^20–23^

**Fig. 1:**
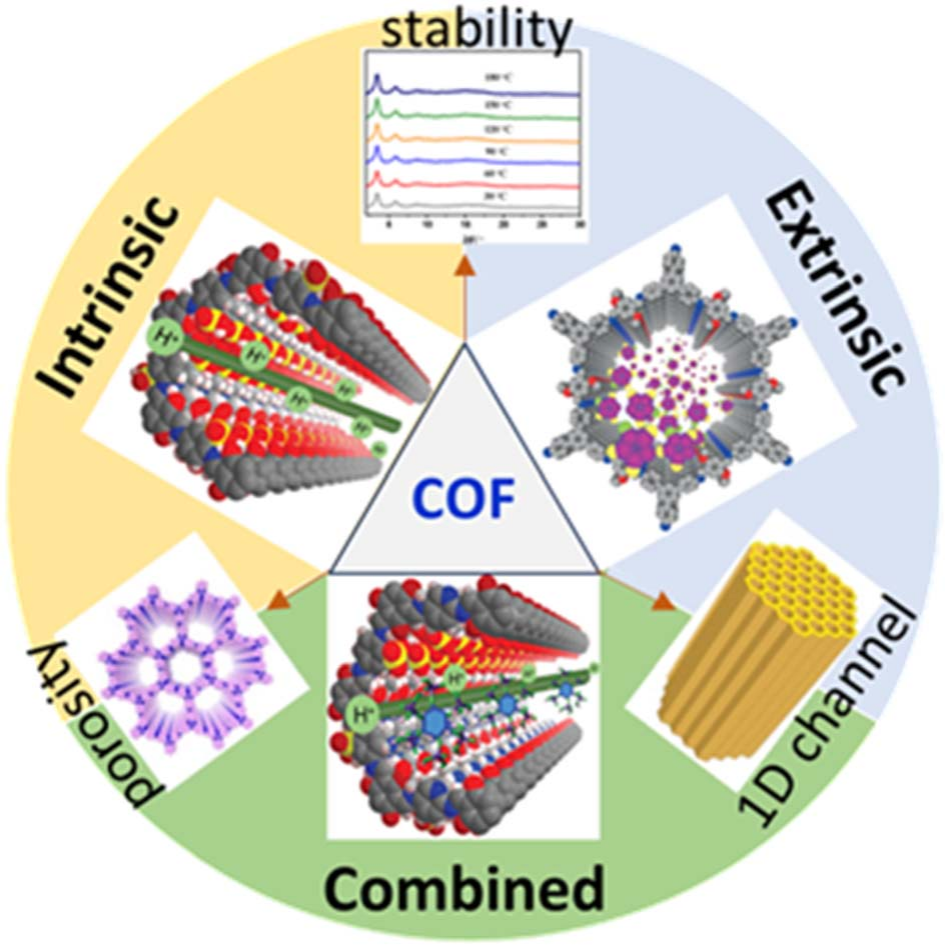
Schematic illustration of characteristics of COFs and their proton conducting mechanism. Porous structures are reproduced with permission from ref. 52, 57, 62 and 65.

Thus far, commercially available perfluorinated sulfonated polymer (Nafion) has been dominantly explored as proton conducting membranes in fuel cells.^24^ However, high cost of synthesis and steady decline of ionic conduction at operation condition of fuel cells such as higher temperature (over 120 °C) and lower RH (<50% RH) restrict their viability for large scale commercial applications.^25,26^ Further, higher humidity decomposes the Nafion membrane over the period of time owing to variable temperature. In this scenario, COFs emerged as a potential candidate for solid-state electrolyte materials in proton conducting membrane fuel cells. Many COF materials account for high proton conductivity on par with that of commercial Nafion. In general, COFs are considered as electrical insulators.^27^ So, COFs are doped with proton sources like H_3_PO_4_, H_2_SO_4_, ionic liquids, *etc.* to ensure the availability and efficient conduction of protons in their skeletons.^28–33^ As a result, proton conduction in 1D pathway along the pores of COF skeleton is made possible. In addition, COFs as solid electrolytes selectively conduct protons from anode to cathode in fuel cells. Further, COFs do not allow the passage of electrons across the membrane and thus act as electrical insulators. Also, the well-defined porosity and 1D channels of COFs allow them to achieve these demanding properties. These unique characteristics attracted to deploy them in fuel cells as solid electrolytes. Generally, amorphous COFs results in poor proton conduction.^34,35^ Thus, it is important to retain crystallinity of COFs for higher proton conduction. So, the precise synthesis of crystalline COFs is dominated for achieving excellent proton conduction and to understand proton transport mechanism at molecular level.^8,36–38^ However, proton conductivity falls steeper after a short period of time owing to the leaching of acids (proton sources). To circumvent this limitation, choice of functional group present on COF and external proton source are to be chosen to tune the effective locking and hopping of proton. Nevertheless, proton conduction in such COFs is dependent on the humidity of environment which restricts their application in high temperature fuel cells especially at harsh conditions.^39^ In addition, they do not exhibit appreciable intrinsic proton conductivity for the practical applications at elevated temperature.^40–43^ Moreover, low-temperature proton exchange membrane fuel cells suffer low electrode kinetics, catalyst toxicity and complex water management. In order to overcome these limitations, high-temperature proton exchange membranes that could exhibit stable proton conduction over subzero temperatures to elevated temperatures are in high demand.^44–48^ Thus, COFs exhibiting excellent proton conduction over 1 × 10^−1^ S cm^−1^ under anhydrous condition are actively researched. In general, H_3_PO_4_ is widely used as proton conducting carrier in COFs owing to its non-volatile and non-toxic characteristics. H_3_PO_4_ could form dense hydrogen bonding network *via* P=O⋯H–O and P–O⋯H–O interactions along 1D pores of COFs and thereby attain high proton conduction. Also, H_3_PO_4_ possesses high boiling point (∼158 °C) and thus expected to exhibit stable proton conduction under anhydrous condition at elevated temperature. Till date, proton conducting COFs under anhydrous condition is not exclusively reviewed. We attempted to compile anhydrous proton conducting COFs reported so far and comprehensively understand their proton conduction behavior by drawing structure–property relationships. In this review, we present the progressive development of COFs doped with H_3_PO_4_/triazole/imidazole/phytic acid/ionic liquids (IL) exhibiting high proton conduction under anhydrous conditions potential for fuel cell applications. Further, we discuss the comprehensive development of COFs displaying anhydrous proton conduction, current challenges and their future prospects in electrochemical energy devices (Fig. 1).

## Intrinsic proton conducting COFs under anhydrous conditions

**Fig. 2:**
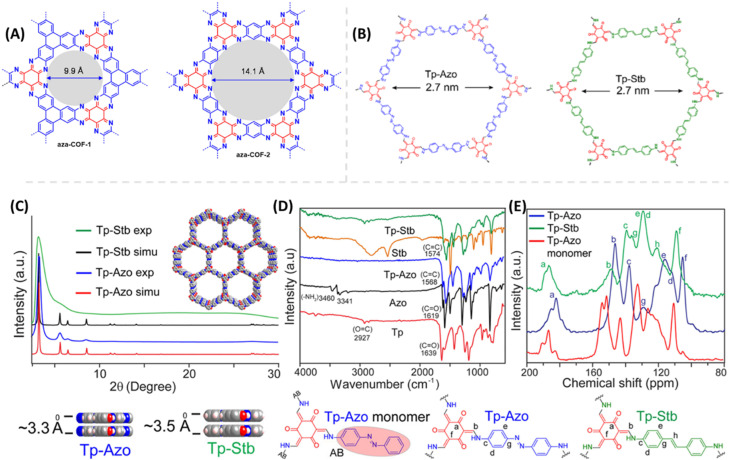
(A) Chemical structure of aza-COF-1 and aza-COF-2. Reprinted with permission,^49^ Copyright (2019) from American Chemical Society. (B) Chemical structure of Tp-Azo and Tp-Stb. (C) Simulated and experimental PXRD pattern of Tp-Azo & Tp-Stb. (D) FTIR spectra of Tp-Azo, Tp-Stb and their monomers. (E) ^13^C NMR spectra of Tp-Azo & Tp-Stb with reference to Tp-Azo monomer (red). Reprinted with permission,^50^ Copyright (2014) from American Chemical Society.

Proton conduction in COFs generally takes place through intrinsic proton conducting groups present in their skeleton or external proton source doped with COFs nano pores. Mostly intrinsic proton conducting COFs exhibit lower conductivity under harsh conditions makes them undesirable for practical applications. For example, Mirica and coworkers^49^ developed proton conducting 2D aza fused π conjugated COFs as proton conducting materials (Fig. 2A). Despite of high good proton conductivity under humidified condition, they showed very low proton conductivity of about 10^−8^ to 10^−9^ S cm^−1^ under anhydrous condition at 50 °C. In most of intrinsic proton conducting COFs, the observed conductivity is very low. So, the external proton source is doped to boost their proton conductivity. Further, the energy of activation (*E*_a_) plays an important role in proton conductivity which is determined experimentally by electrochemical impedance spectroscopy (EIS) measurements. The lower *E*_a_ facilitates the higher proton conductivity. If *E*_a_ is less than 0.40 eV, the reaction mechanism is defined as Grotthuss mechanism in which migration of proton occurs along the hydrogen bonding network. On the other hand, if *E*_a_ is greater than 0.40 eV, proton conduction occurs *via* vehicular mechanism where the migration of proton takes place along with the proton source.

## Extrinsic proton conducting COFs under anhydrous conditions

COFs doped with external proton source show considerably higher proton conduction even at high temperature (>120 °C). In principle, the integration of proton conducting carriers into the porous sites of COF leads to the activation of proton flow by the interaction between proton carriers and porous materials. In case of weak or no interaction between proton carriers and porous materials, it would result in leakage of ion carriers and thus compromise the stability of COFs as well as the fuel cell device. H_3_PO_4_ is widely used as dopant for COFs owing to their resulting high proton mobility stemmed from extended H-bonding from three ionizable O–H bonds and low volatility (high boiling temperature ∼158 °C) which guarantees an uninterrupted operation at higher temperature. Also, we summarize COFs doped with other external proton sources exhibiting anhydrous proton conduction. Moreover, the pronounced effect of external proton source on the proton conduction of intrinsic COFs is also described.

## H_3_PO_4_ doped COFs as anhydrous proton conducting materials

Banerjee and coworkers^50^ first demonstrated azo-functionalized COFs (Tp-Azo and Tp-Stb) as proton conducting material by loading H_3_PO_4_ into the pores of COFs (Fig. 2B). The loading of H_3_PO_4_ was carried out by immersing the COF materials into phosphoric acid over 2 h followed by washing with plenty of water and then activating them overnight at 353 K under dynamic vacuum condition. Phosphoric acid (PA) doped Tp-Azo and Tp-Stb were denoted as PA@Tp-Azo and PA@Tp-Stb respectively. PA@Tp-Azo exhibited very good proton conduction over stilbene counterpart (PA@Tp-Stb) which is ascribed to protonation of azo groups and followed stabilization of counter anion, dihydrogen phosphate (H_2_PO_4_^−^). Besides, the interaction of H_3_PO_4_ with Tp-Azo COF was confirmed by measuring UV-vis spectra of Tp-Azo monomer treated with H_3_PO_4_ which showed red shift in absorption maximum from 380 to 496 nm owing to protonation of azo bond. PA@Tp-Azo showed the proton conductivity of 6.7 × 10^−5^ S cm^−1^ under anhydrous condition at 340 K. However, the proton conductivity was increased as the humidity increases and reaches the maximum value of 9.9 × 10^−4^ S cm^−1^ at 332 K and 98% RH. But, PA@Tp-Stb gave almost zero conductivity under anhydrous conditions while slight conductivity of 2.3 × 10^−5^ S cm^−1^ at 332 K and 98% RH. Moreover, these COF materials were chemically stable in harsh acidic conditions after impregnation of acids and retained their crystallinity.

**Fig. 3:**
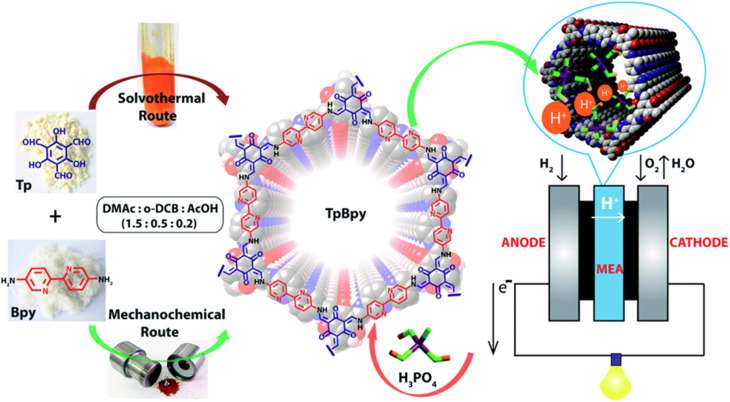
Synthesis of TpBpy COF *via* solvothermal and mechanochemical routes, doping of H_3_PO_4_ and their implementation in fuel cell as solid electrolyte. Reprinted with permission,^51^ Copyright (2016) from Royal Society of Chemistry.

Generally, COFs are synthesized by solvothermal methods. However, mechanochemical synthesis is considered to be cost-effective, and eco-friendly compared to solvothermal synthesis, but predominantly results in poor crystallinity. Banerjee's group attempted mechanochemical synthesis of COF (TpBpy-MC) containing bipyridine units that exhibit excellent proton conduction and high crystallinity (Fig. 3).^51^ The mechanochemical synthesis was carried out by taking 1,3,5-triformylphloroglucinol (0.3 mmol), 2,2′-bipyridine-5,5′-diamine (0.45 mmol), *N*,*N*′-dimethylacetamide (DMAc) 60 μL, *o*-dichlorobenzene (30 μL), 6 M AcOH (15 μL) in a stainless steel jar with one 7 mm stainless steel ball and ball milled with different milling time and frequency. The optimized reaction condition is 30 Hz and grinding time of 90 minutes to obtain TpBpy-MC. On the other hand, solvothermal synthesis was carried out by charging 1,3,5-triformylphloroglucinol (0.3 mmol), 2,2′-bipyridine-5,5′-diamine (0.45 mmol), *N*,*N*′-dimethylacetamide (4.5 mL), *o*-dichlorobenzene (1.5 mL), 6 M AcOH (0.6 mL) in a tube. They mixed the reactants homogeneously by ultrasonication for 15 minutes followed by degassing through three successive freeze–pump–thaw cycles. Then, the tube was vacuum sealed and heated for 72 h at 120 °C. After cooling to room temperature, the reaction mixture was solvent exchanged with DMAc and washed with excess of water and acetone. Further, the material was dried under vacuum at 150 °C for 12 h to obtain the COF, TpBpy-ST. Bipyridine unit present in the COF is potentially beneficial to immobilize H_3_PO_4_ by forming hydrogen bonding and thereby migration of proton through hydrogen bond networks along the 1D nanopores. The proton conductivity of H_3_PO_4_ doped mechanically synthesized COF (H_3_PO_4_@TpBpy-MC) outperformed its solvothermally synthesized counterpart (TpBpy-ST) under similar operating conditions. Besides, the elevation of proton conductivity was observed as the temperature rose from −40 to 120 °C. Notably, the maximum proton conductivity of 2.5 × 10^−3^ S cm^−1^ and 1.98 × 10^−3^ S cm^−1^ was observed at 120 °C, under anhydrous condition for PA@TpBpy-MC and PA@TpBpy-ST respectively. Further, solid-state electrolyte conductivity of 1.4 × 10^−2^ S cm^−1^ at 50 °C was observed for PA@TpBpy-MC. Moreover, membrane electrode assembly (MEA) fabricated with PA@TpBpy-ST showed open circuit voltage (OCV) of 0.66 ± 0.02 V at 50 °C while PA@TpBpy-MC gave the OCV of 0.92 V, maximum current density of 29 mA cm^−2^ and maximum power density of 7 mW cm^−2^.

**Fig. 4:**
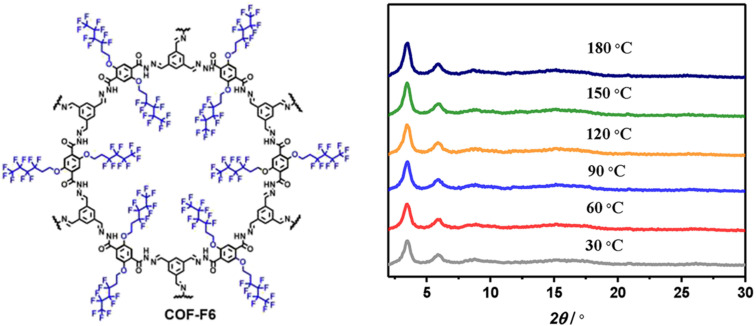
Structure of COF-F6 and the corresponding PXRD pattern at various temperature. Reprinted with permission,^52^ Copyright (2020) from American Chemical Society.

Horike and coworkers synthesized superhydrophobic perfluoroalkyl substituted hydrazone linked 2D COFs as anhydrous proton conductors (Fig. 4).^52^ All the fluorinated COFs showed high water contact angle of 144° which is higher than that of polytetrafluoroethylene (PTFE, 120°). Further, alkyl fluorinated COFs exhibited higher acid resistance over non-fluorinated COFs owing to elevation of hydrophobicity. The unaltered PXRD peak pattern of COF-F6 over the temperature range of 30–180 °C indicates the retention of crystalline nature which is beneficial for achieving anhydrous proton conductivity at elevated temperature (Fig. 4). The abundant –NH group present in COF induced the formation of numerous hydrogen bonding with H_3_PO_4_ through interaction between P=O⋯H–N bond. In addition, H_3_PO_4_ doped COF-F6 exhibited downshifted ^1^H NMR chemical shift for NH proton from 10 to 11.2 ppm which reinforces the above statement. Moreover, the increasing amount of H_3_PO_4_ results in progressive result in proton conductivity and maximum conductivity of 4.2 × 10^−2^ S cm^−1^ observed for 62 wt% of H_3_PO_4_@COF-F6 at 140 °C under anhydrous condition. Interestingly, highly stable proton conductivity of 1.65 × 10^−2^ S cm^−1^ was recorded for 42 wt% H_3_PO_4_@COF-F6 and remains stable up to 40 h at 140 °C. However, non-fluorinated COF resulted in about 4 orders lower magnitude of proton conductivity over fluorinated counterpart. The ‘N’ sites and CF_2_ present in COF pores were helpful to align H_3_PO_4_ network in one direction and thereby reaches high proton flow.

**Fig. 5:**
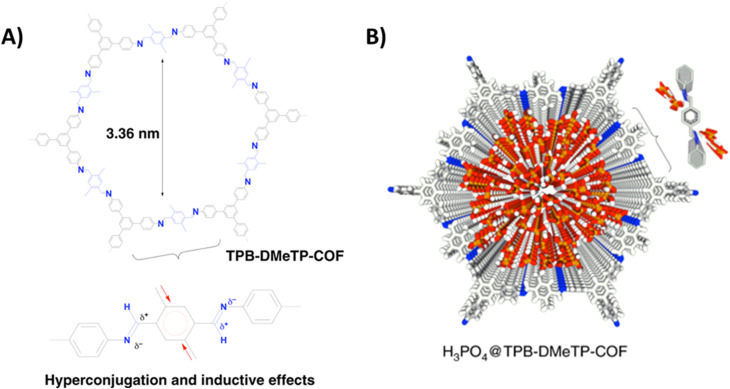
(A) Structure of TPB-DMeTP-COF. Inset indicates the inductive and hyperconjugation effects of methyl groups that reduces the polarisation of C=N bonds, softens interlayer charge repulsion and yields an extremely stable framework. (B) Reconstructed crystal structure of H_3_PO_4_@TPB-DMeTP-COF (top view) with the H_3_PO_4_ network locked in the 1D channel (each macrocycle of one layer contains 57 H_3_PO_4_ molecules; only 20 layers are shown; grey, C; blue, N; white, H; red, O; orange, P). Reprinted with permission,^53^ Copyright (2020) from Nature publications.

Jiang and coworkers reported imine-bonded COF (TPB-DMeTP-COF) possessing dense and one dimensionally aligned nanochannels (Fig. 5).^53^ It was demonstrated that the presence of methyl group in phenyl linker assisted to achieve stable pore structure through hyperconjugation and inductive effect, while nitrogen sites on pore wall guarantee confinement and stabilization of H_3_PO_4_ network through hydrogen bonding interactions. Each COF unit have six nitrogen atoms (imine-bonded) available for formation of hydrogen bonding with H_3_PO_4_*via* N⋯H–O interactions which eventually extended to three-dimensional multi-chain multipoint mode. Thus, TPB-DMeTP-COF stabilized its own pore structure as well as H_3_PO_4_ network along the channels. Notably, this COF is stable under harsh conditions (strong acid, strong base, water and organic solvents like THF, ACN) for 7 days and retained its crystallinity and porosity. H_3_PO_4_ was loaded into the COF to prepare H_3_PO_4_@TPB-DMeTP-COF by vacuum impregnation method and found to have phosphoric acid content of 266.6 wt% which is almost near to the maximum loading content (269.6 wt%). H_3_PO_4_@TPB-DMeTP-COF exhibited proton conductivity of 1.91 × 10^−1^ S cm^−1^ which is almost double of molten neat H_3_PO_4_ (≈1 × 10^−1^ S cm^−1^). Interestingly, proton conduction is stable for 20 h at 160 °C under anhydrous conditions. Also, proton conductivity increased as the temperature is raised from 100 to 160 °C at the interval of each 10 °C. Moreover, reduced proton conduction was observed when H_3_PO_4_ loading content is decreased. However, the magnitude of proton conductivity is higher than that of polybenzimidazoles which is attributed to dense nitrogen sites (imine-bonded) in pore walls that lead to continuous H_3_PO_4_ network at less amount of H_3_PO_4_ loading.^54,55^ In addition, the proton diffusion rate calculated from molecular dynamics at picosecond level was 6.28 × 10^−4^ cm^2^ s^−1^ at 383 K which is much higher than that for neat H_3_PO_4_ film (4.0 × 10^−6^ cm^2^ s^−1^) under same conditions. It is shown that high proton diffusion rate and the less reorganization energy of H_3_PO_4_ (0.12 eV) resulted in super flow of protons.

**Fig. 6:**
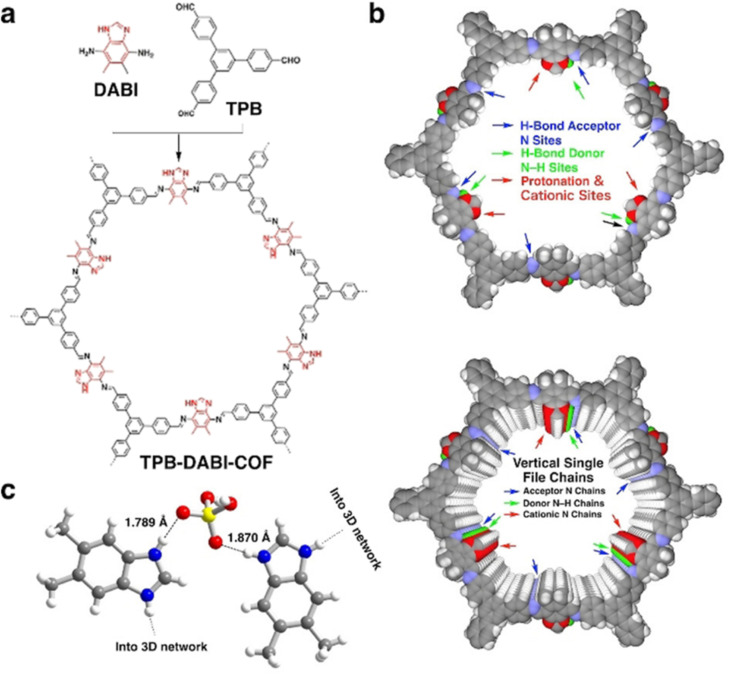
(a) Structure of polybenzimidazole COF. (b) Reconstructed structures of a single layer (top panel) and ten layers (bottom panel) of the TPB-DABI-COF and sites and vertical single file chains to confine and activate the H_3_PO_4_ network. (c) Single-crystal structure of the benzimidazole–H_3_PO_4_ complex shows N–H⋯O hydrogen-bond and electrostatic interactions; one N of the imidazole ring is protonated by H_3_PO_4_. Atom key: N (blue), O (red), H (white). Reprinted with permission,^56^ Copyright (2021) from Wiley.

Later, Jiang and coworkers reported polybenzimidazole based COF namely TPB-DAPI-COF (Fig. 6).^56^ In contrast to imine linked COF, benzimidazole linked COF exhibited higher proton conductivity under anhydrous conditions. Interestingly, benzimidazole chains present in the COF activates electrostatic and hydrogen bonding interactions with H_3_PO_4_ network and thereby stabilize and lock the H_3_PO_4_ network in COF by forming H_3_PO_4_–benzimidazole complex. Single crystal X-ray diffraction studies confirmed the interaction between imidazolium cation and deprotonated H_2_PO_4_^−^ which is beneficial for facile proton transport (Fig. 6c). Generally, polybenzimidazole system possesses low proton conductivity compared to neat H_3_PO_4_ neat film, owing to steric hindrance in polybenzimidazoles restricts the motion of proton and swollen chain permits the leakage of H_3_PO_4_. However, the highest proton conductivity of 1.51 × 10^−1^ S cm^−1^ was observed for H_3_PO_4_@TPB-DAPI-COF at 160 °C. Interestingly, H_3_PO_4_ loading is only 66.1% which is one fourth of H_3_PO_4_ content (loading capacity of 226.6 wt%) in H_3_PO_4_@TPB-DMeTP-COF (Fig. 5) that showed the proton conductivity of 1.91 × 10^−1^ S cm^−1^ at 160 °C.^53^ Furthermore, H_3_PO_4_@TPB-DAPI-COF showed no decline in proton conductivity at 160 °C and retained its initial value for 120 h. The activation energy for H_3_PO_4_@TPB-DAPI-COF is 0.17 eV which is about half of H_3_PO_4_@TPB-DMeTP-COF (0.34 eV) which supported high rate of proton conduction. It is worth noting that H_3_PO_4_@TPB-DAPI-COF exhibited higher proton conductivity over H_3_PO_4_@TPB-DMeTP-COF at 100–130 °C. The amorphous counterpart of TPB-DAPI was also synthesized and found to exhibit lower proton conductivity which is due to higher activation energy (0.61 eV) and irregular pores that suppress the extension of hydrogen bonding H_3_PO_4_ network.

**Fig. 7:**
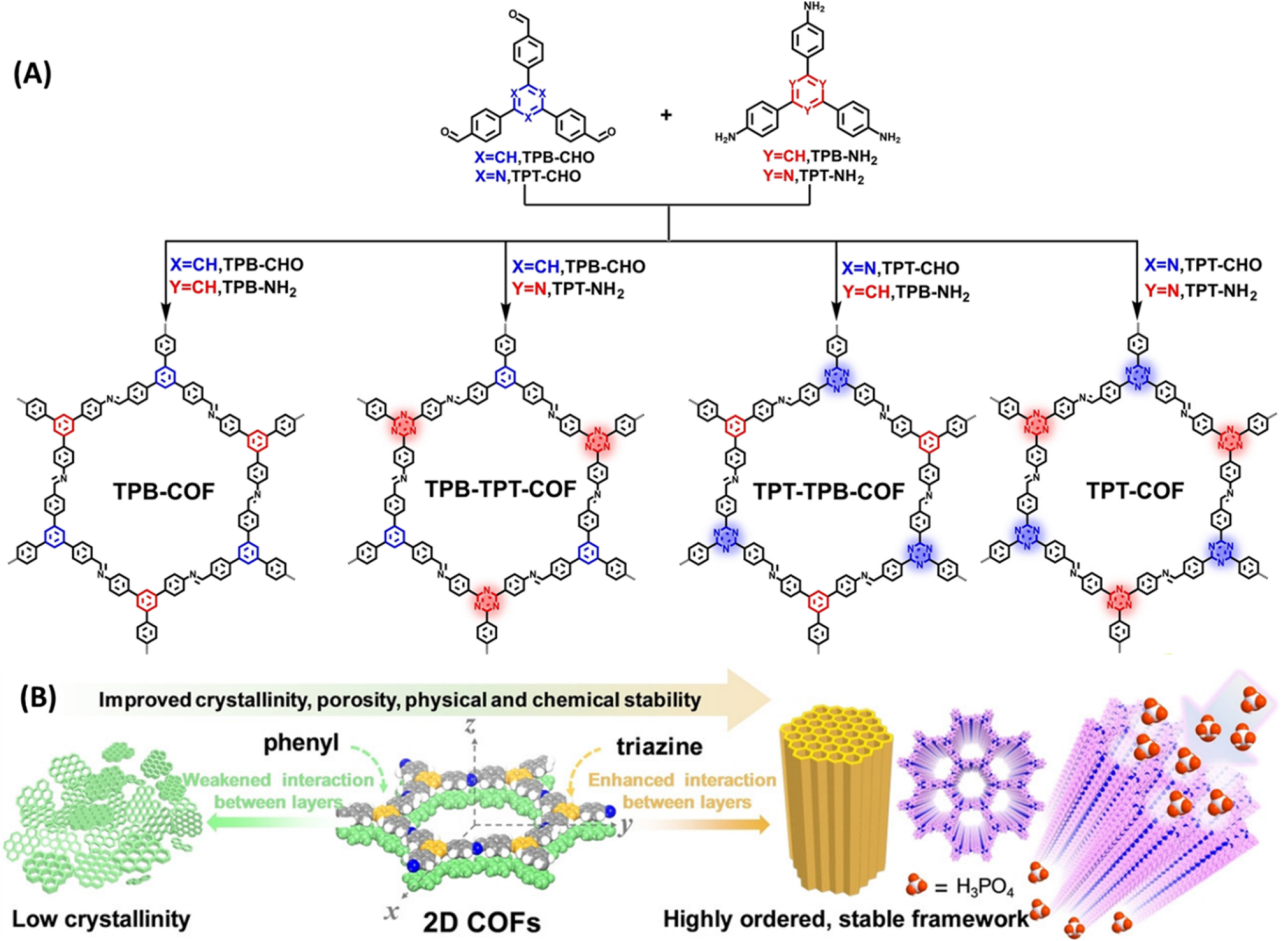
(A) Bottom-up strategy for the synthesis of TPB-COF, TPB-TPT-COF, TPT-TPB-COF, and TPT-COF. (B) Schematic illustration of the crystallite growth controlled by π–π stacking interactions between layers. Reprinted with permission,^57^ Copyright (2022) from Wiley.

COFs displaying poor chemical stability owing to weak interaction between the layers and the intrinsic reversibility of linkages such as imine bond are need to be carefully engineered to yield highly stable COFs. Du and coworkers developed imine and triazine based COFs and compared their proton conducting properties (Fig. 7).^57^ They demonstrated that triazine units present in COF is desirable for having strong interaction with H_3_PO_4_ and thereby suppress the reversibility of C=N (imine) bonds and proton leakage. The arrangement of nitrogen atoms on pore walls facilitated the formation of dense H_3_PO_4_ networks and thus resulted in high proton conduction. COFs possessing irreversible triazine units showed strong interaction between the layers compared to their phenyl counterpart and thereby emerged as highly ordered crystalline materials (Fig. 7B). TPT-COF showed highly ordered stable frameworks owing to six triazine unit present in the single pore structure which was ascertained from the lowest full width at half maximum (FWHM) of diffraction (100) value of 0.43° among the four COFs. However, the FWHM (100) value for TPB-COF, TPB-TPT-COF, TPT-TPB-COF are to be 0.83°, 0.71°, 0.59° and thus TPB-COF exhibited poor induced interlayer stacking and low crystallinity. Interestingly, unaltered PXRD pattern confirmed the excellent chemical stability and crystallinity of TPT-COF after soaked in 14.6 M H_3_PO_4_ for two months. Further, TPT-COF exhibited anti-oxidation stability against Fenton's reagent after 24 h immersion. The lowest inter-layer stacking distance and high π–π stacking energy indicates the strong interlayer interaction for TPT-COF. They loaded 85% H_3_PO_4_ into TPT-COF and the calculated loading content of H_3_PO_4_ was 65.4%. H_3_PO_4_@TPT-COF showed the highest proton conductivity of 1.27 × 10^−2^ S cm^−1^ at 160 °C with negligible change in conductivity for over 100 h. Also, the proton conductivity increased as the temperature rose from 100–160 °C. In addition, H_3_PO_4_ loading content also played a crucial role in proton conduction. As the loading content increased from 18 to 65%, proton conductivity also raised from 8.84 × 10^−5^ to 1.27 × 10^−2^ S cm^−1^. Similarly, activation energy was decreased as the H_3_PO_4_ loading content increased which further attests the higher proton conductivity at higher loading content of H_3_PO_4_ in the pore channel. The synergistic effect of triazine nitrogen and imine nitrogen on stabilization of H_3_PO_4_ network in COF pore walls resulted in high proton conductivity with long durability.

**Fig. 8:**
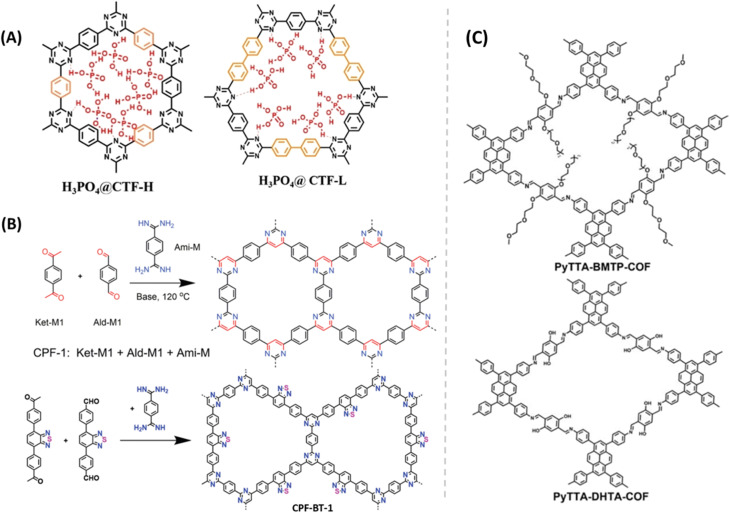
(A) Illustration of interaction of H_3_PO_4_ in the pores of CTF-H and CTF-L. Reprinted with permission,^58^ Copyright (2022) from Wiley. (B) Structure of CPF-1 and CPF-BT-1. Reprinted with permission,^59^ Copyright (2023) from Wiley. (C) Structure of pyrene-based COFs. Reprinted with permission,^60^ Copyright (2023) from Royal Society of Chemistry.

Mostly, H_3_PO_4_@COF was successfully demonstrated as excellent proton conductors at high humidity. However, H_3_PO_4_@COFs as proton conductors at anhydrous and higher temperature under open air conditions, (extreme conditions) are scarcely studied. Liu *et al.* synthesized triazine based COF (CTF-H) and impregnated H_3_PO_4_ into it (Fig. 8A).^58^ The resulting COF exhibited high proton conductivity of 1.6 × 10^−1^ S cm^−1^ at elevated temperature of 150 °C (0% RH). Interestingly, this high conductivity lasts for 18 months under open air conditions which is important for practical applications. The high proton conductivity of the COF may be attributed to fast formation and breaking of hydrogen bond between nitrogen of triazine unit and OH– group of phosphoric acid (Ntriazine⋯H^+^⋯H_2_PO_4_^−^ pairs). Moreover, the conductivity is higher than that of neat phosphoric acid (about 1 × 10^−1^ S cm^−1^). In contrast, H_3_PO_4_@CTF-L showed proton conductivity of 5.1 × 10^−2^ S cm^−1^ under same operating conditions which is due to the lower number of pyridinic nitrogen and the generated activated Ntriazine⋯H^+^⋯H_2_PO_4_^−^ pairs (*i.e.*, lower number of proton carriers). Nevertheless, the pristine CTF-H (non-doped) showed low proton conductivity in the range of 2.6 × 10^−7^ to 3.5 × 10^−7^ S cm^−1^ at temperature range of 30–150 °C, under anhydrous conditions which might be due to lack of free H^+^ carriers in the COF frameworks. The activation energy of CTF-H and CTF-L is to be 0.248 and 0.259 eV which indicates the Grotthuss-type mechanism for proton conduction. However, the absence of water at higher temperature (120–150 °C) supported the proposed proton conduction mechanism *via* activated Ntriazine⋯ H^+^⋯H_2_PO_4_^−^ pairs.

Jin and coworkers reported pyrimidine-based COFs (CPF-1 and CPF-BT-1) using an efficient tandem polycondensation reaction between aldehyde, acetyl and amidine under mild condition (Fig. 8B).^59^ Both COFs exhibited high thermal stability (>450 °C). They prepared mixed matrix membranes by embedding these COFs (40%) into polyvinylidene fluoride (PVDF). H_3_PO_4_ doped mixed membranes were analyzed for proton conductivity over 100–160 °C. At 140 °C, the maximum proton conductivity of 1.30 × 10^−2^ and 9.77 × 10^−3^ S cm^−1^ was observed for H_3_PO_4_@CPF-BT-1/PVDF and H_3_PO_4_@CPF-1/PVDF respectively.

Zeng and coworkers developed pyrene-based 2D COFs having dense H-bond acceptors in pore channels to promote proton conduction under anhydrous conditions (Fig. 8C).^60^ They manipulated the number of oxygen atoms (H-bond acceptors) in the porous site to modulate their proton conductivity. PyTTA-BMTP-COF possesses higher number of oxygen atom (12 O atoms) over PyTTA-DHTA-COF (4 O atoms) on its pores. PA@PyTTA-BMTP-COF exhibited high proton conductivity of 2.6 × 10^−2^ S cm^−1^ while PA@PyTTA-DHTA-COF showed 9.2 × 10^−3^ S cm^−1^ at 140 °C under anhydrous condition. The highest proton conductivity of PA@PyTTA-BMTP-COF is attributed to high density of available oxygen atoms in COF pores to interact with H_3_PO_4_ for the formation of hydrogen bonds and thus facilitated proton conduction.

**Fig. 9:**
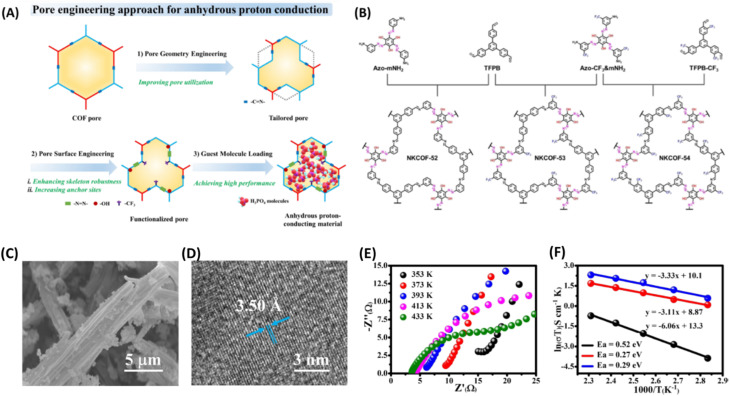
(A) Illustration of pore engineering approach for the construction anhydrous proton conducting COF. (B) Schematic synthetic approach for the NKCOFs. (C) SEM image of NKCOF-52. (D) HR-TEM image of NKCOF-52. (E) Nyquist plots of H_3_PO_4_@NKCOF-54 measured at different temperatures. (F) Arrhenius plots for H_3_PO_4_@NKCOF-52 (black), H_3_PO_4_@NKCOF-53 (red) and H_3_PO_4_@NKCOF-54 (blue). Reprinted with permission,^61^ Copyright (2023) from Wiley.

More recently, Zhang and coworkers^61^ manipulated the pore size and pore surface of COFs (NKCOF-52, -53, 54) decorated with choice of functional groups to realize anhydrous proton conduction over wide range of temperature from 80–160 °C (Fig. 9). The azo groups act as effective sites for locking H_3_PO_4_ and phenolic hydroxy group ensures intrinsic proton conduction, while CF_3_ establishes hydrophobicity and high stability of NKCOFs. At 160 °C, the highest anhydrous proton conductivity of 1.12 × 10^−3^, 1.24 × 10^−2^ and 2.33 × 10^−2^ S cm^−1^ was observed for NKCOF-52, NKCOF-53 and NKCOF-54 respectively. However, other synthesized COFs having similar pore size but without azo- and phenyl hydroxy groups resulted in lower proton conduction. It is interesting to note that the anhydrous proton conductivity of NKCOF-53 and NKCOF-54 were to be 4.51 × 10^−3^ and 1.26 × 10^−2^ S cm^−1^ at 160 °C for the same amount of H_3_PO_4_ doping which demonstrate the influence of functional groups present on pore walls.

## Phytic acid doped COFs as anhydrous proton conducting materials

**Fig. 10:**
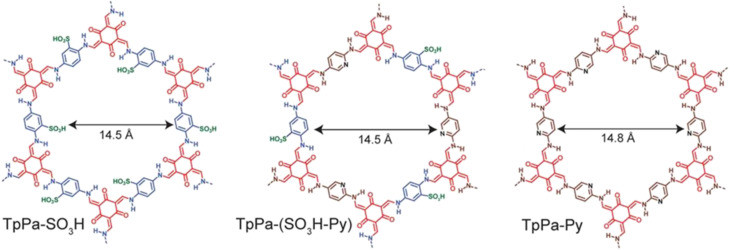
Structure of TpPa-SO_3_H, TpPa-(SO_3_H-Py) and TpPa-Py. Reprinted with permission,^62^ Copyright (2016) from American Chemical Society.

Banerjee's group reported COFs possessing intrinsic proton conducting groups (sulfonic groups in TpPa-SO_3_H) or basic groups that immobilize guest molecules for facile proton conduction (pyridine in TpPa-Py) (Fig. 10).^62^ Also, they prepared COF having combination of both intrinsic and extrinsic conducting groups, TpPa-(SO_3_H-Py). TpPa-SO_3_H exhibited the highest conductivity of 1.7 × 10^−5^ S cm^−1^ at 120 °C. However, TpPa-Py and TpPa-(SO_3_H-Py) showed no conductivity under the same conditions. Phytic acid exhibiting low volatility and well-matched dimensions with COF pore size was doped in to these COFs to prevent leaching of acid and to enhance proton conductivity. Phytic@TpPa-(SO_3_H-Py) showed better conductivity of 5 × 10^−4^ S cm^−1^ than phytic@TpPa-SO_3_H and phytic@TpPa-Py at 120 °C under anhydrous conditions. The higher proton conductivity of phytic@TpPa-(SO_3_H-Py) was ascribed to both intrinsic conduction from sulfonic acid groups and extrinsic conduction from pyridine groups that maintain immobilization of phytic acid inside COF pores.

## PIL doped COFs as anhydrous proton conducting materials

**Fig. 11:**
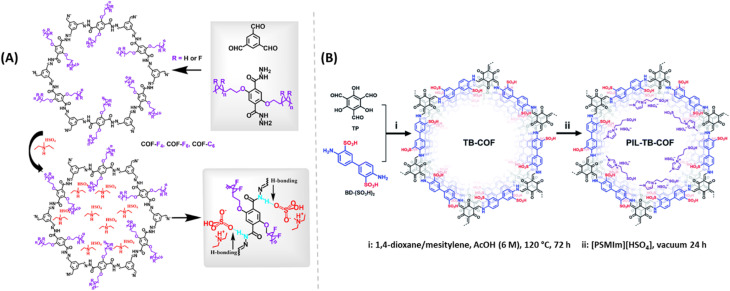
(A) Synthesis of alkyl/perfluoroalkyl functionalized COFs doped with PILs and the hydrogen bond networks demonstrated. Reprinted with permission,^63^ Copyright (2021) from American Chemical Society. (B) Synthesis of PIL doped TB-COF. Reprinted with permission,^64^ Copyright (2022) from Royal Society of Chemistry.

Zhang and coworkers developed alkyl-functionalized 2D COFs (Fig. 11A). Among them, perfluoroalkyl functionalized COF showed excellent thermal and chemical stability.^63^ They impregnated two different protic ionic liquids (PILs), dema[HSO_4_] and dema[H_2_PO_4_] into these COF materials by pressing them into pellets under Ar atmosphere in a glove box. After doping, these COFs retained their crystallinity. COF-C6 doped with dema[HSO_4_] showed about 2 order higher magnitude of proton conductivity compared to COF-C6 doped with dema[H_2_PO_4_] dopant at 1 : 1 weight ratio over the range of temperature from 40–140 °C. As expected, amount of doping pronounced a positive effect on the proton conductivity. At higher weight doping, activation energy is reduced which resulted in higher proton conductivity. COF-F6 doped with dema[H_2_PO_4_] at equal weight ratio maintained the proton conductivity for 48 h at 120 °C. Also, the highest proton conductivity of 1.33 × 10^−2^ S cm^−1^ was recorded at the weight ratio of 1 : 1.5 for COF-F6-[dema-HSO_4_].

Also, Yan and coworkers demonstrated the incorporation of PILs to enhance the proton conduction of TB-COF (Fig. 11B).^64^ The tailored conducting groups (–SO_3_H groups) on TB-COF induced the highest inherent proton conductivity of 1.52 × 10^−4^ S cm^−1^ at 120 °C. PIL is a combination of Brønsted acid and base that can form hydrogen bond network and behaves as proton carriers. After doping with PIL 1-methyl-3-(3-sulfopropyl)imidazolium hydrogensulphate ([PSMIm][HSO_4_]), into TB-COF, the resulting PIL-TB-COF gave enhanced proton conductivity to 2.21 × 10^−3^ S cm^−1^ at 120 °C under anhydrous condition, due to the electrostatic interaction between PIL and TB-COF. The lower *E*_a_ for PIL-TB-COF over TB-COF indicates the role of PIL in reduction in energy barrier for H^+^ transfer and thereby improved proton conductivity under similar conditions. Both the intrinsic and extrinsic conduction of sulphonic acid group of COF skeleton and PIL synergistically enhanced the proton conduction. Moreover, PIL-TB-COF retained its proton conductivity almost unaltered over 72 h at 120 °C which is advantageous for practical applications.

## Imidazole-doped COFs as anhydrous proton conductors

**Fig. 12:**
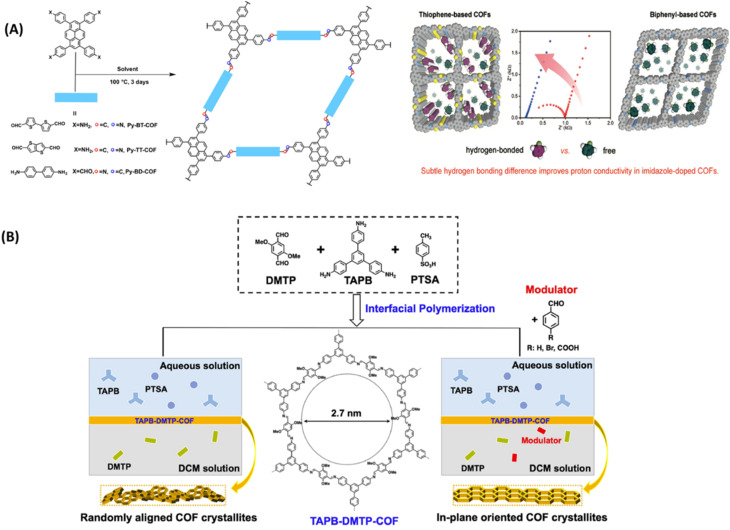
(A) Synthesis of COFs and the demonstrated hydrogen bonding between doped imidazole with thiophene-based COFs. Reprinted with permission,^65^ Copyright (2020) from American Chemical Society. (B) Synthesis of TAPB-DMTP-COF by interfacial polymerization. Reprinted with permission,^66^ Copyright (2022) from Springer.

Anhydrous proton exchange membranes (PEM) offer viability for operation at elevated temperatures (120–200 °C), which is advantageous for fast electrode reaction, and higher tolerance to CO poisoning over Pt catalyst. In general, the presence of azo, pyridine, azine and sulfone groups on COFs is exploited as hydrogen bonding sites for proton carriers such as H_3_PO_4_ and imidazole. Wang and coworkers synthesized iso-reticular thiophene-based COF materials (Py-BT-COF, Py-TT-COF, Py-BD-COF) to explore the effect of hydrogen donor ability of imine linkages on proton conductivity (Fig. 12A).^65^ They observed negligible proton conduction for pristine COFs as these COFs do not possess effective proton carriers in the pores. The electron rich thiophene improved the hydrogen bonding of imine linkages and further imidazole doped COF showed improved proton conductivity and lower activation energy. Imidazole was doped into COF in two different ratios such as 30 and 50 wt% by vapor diffusion method. These COFs exhibited high structural stability which is confirmed from nearly unchanged PXRD pattern after proton conduction measurements. The higher loading of imidazole (50 wt%) into COF exhibited reduced activation energy and thus rise in proton conductivity. The highest proton conductivity of 3.08 × 10^−3^ S cm^−1^ was observed for Im@Py-TT-COF(50 wt%) at 130 °C under anhydrous conditions which is about six times higher than that observed for 30 wt% imidazole loaded COF. DFT calculations revealed that the N–H⋯N hydrogen bonding distance between imine and imidazole is 1.932 Å for Im@Py-TT-COF, which is slightly shorter than those observed for Im@Py-BT-COF and Im@Py-BD-COF. This shorter distance and stronger hydrogen bonding was beneficial to well organization of imidazole and their hopping in the pores. Further, this hydrogen bonding was confirmed by the shift in imine stretching frequency of about 8 cm^−1^.

## Triazole doped COFs as anhydrous proton conducting materials

Guo and coworkers developed 2D COFs using in plane crystallization in water/oil interfacial polymerization system *via* reversible aldimine reaction (Fig. 12B).^66^ By addition of modulator into COF membrane, the authors tuned in-plane orientation and large crystalline domains in the COF membranes and thereby tuned proton conductivity. Triazole loaded TAPB-DMTP-COF membrane showed high proton conductivity of 3.10 × 10^−3^ S cm^−1^ at 130 °C under anhydrous conditions. Further, triazine loaded COF containing modulator 4-bromobenzaldehyde (BBA), trz@TAPB-DMTP-COF_0.1-BBA_ membrane gave the highest proton conductivity of 5.05 × 10^−3^ S cm^−1^ which is about 1.6 times higher than that of trz@COF that does not possess BBA units. The lower proton conductivity of trz@TAPB-DMTP-COF membrane is attributed to random orientation which results in inefficient proton conduction. In contrast, anisotropic orientation of 1D pores in trz@TAPB-DMTP-COF_0.1-BBA_ leads to unidirectional proton transport with low activation energy of 0.21 eV and thus resulted in higher proton conductivity.

## PA/triazole doped COFs as anhydrous proton conducting materials

**Fig. 13:**
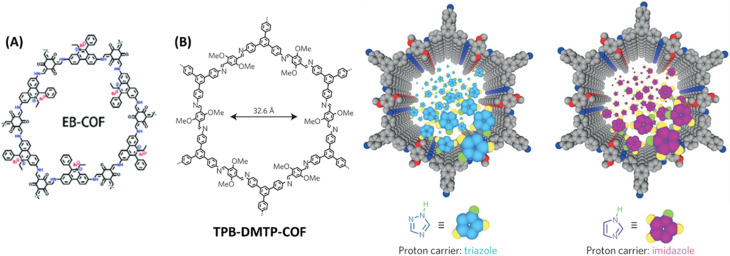
(A) Structure of EB-COF. Reprinted with permission,^67^ Copyright (2020) from Royal Society of Chemistry. (B) Structure of TPB-DMTP-COF and the graphic representation of triazole and imidazole in the channels. Reprinted with permission,^68^ Copyright (2016) from Nature publications.

Generally, proton conductivity in COF depends on the number of free protons available for transportation and well-defined 1D nano channels that ensures the free flow of proton and decrease of resistance. Despite of high proton conductivity of COFs, it is still not comparable to commercial Nafion. It could be due to the high dissociation energy of proton carriers (PA and azoles) in the walls of COF pores compared to that of water in Nafion. Considering this in mind, Ma and coworkers developed four COFs and tuned the proton dissociation energy of proton carriers present in COF and thereby achieved higher proton conductivity.^67^ Phosphoric acid and 1,2,4-triazole have proton dissociation energy of 3.86 and 4.07 eV respectively. After doped into COF, weak interaction between COF skeleton and biphosphate anion (COF⋯H⋯H_2_PO_4_^−^) leads to decrease in proton dissociation energy which eventually results in higher proton conductivity. Particularly, the positively charged EB-COF (Fig. 13A) had strong coulombic interaction with biphosphate anion/triazole. The high proton conductivity of EB-COF could be attributed to ion-pairs present in COF channel that act as proton donor and acceptor, and the resulting formation of extended hydrogen bonds in COF channels. As a result, PA@EB-COF showed higher proton conductivity of 2.77 × 10^−2^ S cm^−1^ at 180 °C under anhydrous condition compared to other COFs under similar condition. Also, for triazole doped COFs, tra@EB-COF gave the maximum proton conduction of 3.25 × 10^−3^ S cm^−1^ at 160 °C under anhydrous condition.

## Triazole/imidazole doped COFs as anhydrous proton conducting materials

Jiang and coworkers developed triazole doped imine-functionalized TPB-DMTP-COF for anhydrous proton conduction at elevated temperature (Fig. 13B).^68^ The retention of crystallinity and porosity of triazole doped TPB-DMTP-COF under harsh conditions such as about 120 °C for 7 days underscores its viability for real time applications. The proton conductivity was dependent on temperature and the maximum conduction recorded was to be 1.1 × 10^−3^ S cm^−1^ at 130 °C under anhydrous condition for trz@TPB-DMTP-COF. Further, triazole/imidazole doped TPB-DMTP-COF stored in 130 °C under sealed condition showed high thermal stability over a period of one month. The highest proton conductivity of im@TPB-DMTP-COF was to be 1.84 × 10^−3^ S cm^−1^ at 130 °C. The lower conductivity of triazole doped COF is attributed to relatively low proton concentration of triazole due to two lone paired nitrogen atoms. Further, deuterium substituted imidazole doped TPB-DMTP-COF confirmed the proton conduction occurs through transport of protons but not the migration of imidazole/triazole molecules.

**Fig. 14:**
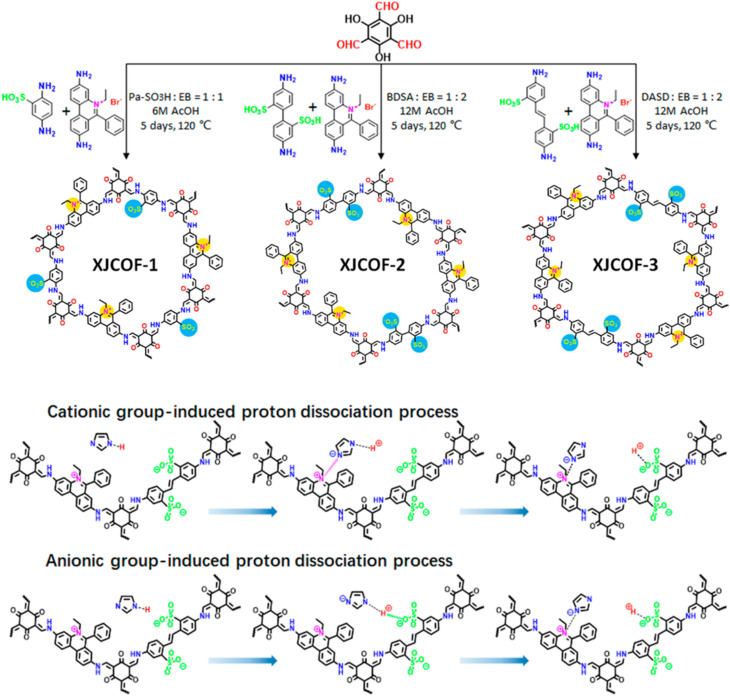
Synthetic scheme of XJCOFs and the two possible processes for inducing proton dissociation in XJCOF-2 skeleton. Reprinted with permission,^69^ Copyright (2021) from American Chemical Society.

Ma and coworkers developed zwitterionic COFs (XJCOF) possessing both anionic and cationic groups in equimolar ratios (Fig. 14).^69^ Three different anilines containing sulfonic acid groups (–SO_3_H) and ethidium bromide were utilized as cationic and anionic monomers respectively, to react with triformylphloroglucinol to obtain three zwitterionic COFs with different pore widths. The resulting COFs obtained in keto form that was confirmed by a peak at 183 ppm (keto group) in cross-polarization angle spinning ^13^C solid state NMR spectrum. Further, the energy dispersive spectroscopy (EDS) confirms the removal of Br^−^ ion of ethidium bromide along with hydrogen of sulfonic acid groups after washing the reaction mixture, which results in neutral COF channel. Triazole/imidazole was incorporated into XJCOF and evaluated the proton conductivity. Particularly, proton conduction is increased as the temperature rise from 120 to 160 °C. The highest proton conductivity of 1.29 × 10^−2^ S cm^−1^ at 160 °C observed for trz@XJCOF-2. Further, im@XJCOFs showed higher proton conductivity over their trz@XJCOF counterparts. The maximum proton conductivities of 4.38 × 10^−2^, 3.33 × 10^−2^, and 2.43 × 10^−2^ S cm^−1^ were observed for im@XJCOF-1, im@XJCOF-2 and im@XJCOF-3 respectively, at 140 °C under anhydrous condition. Moreover, the activation energy of im@XJCOFs was to be about 0.24 eV which is comparable to that of commercial Nafion (0.22 eV). In addition, both anionic and cationic groups present in the zwitterionic COFs could form ion pairs with triazole/imidazole dopant in the nano channels which act as proton donor and acceptor, and thus enhanced the proton diffusion rate.

## Summary and future perspective

Proton conducting covalent organic frameworks (COFs) are promising candidates for the solid-state electrolytes in fuel cells. Compared to other proton conducting materials suffer from low conductivity and poor stability, COFs have uniform 1D channels with permanent porosity for facile transportation of proton carriers and rigid framework-enabled stability. Interestingly, COFs exhibit high robustness towards harsh environments such as strong acid, strong base, and external humidity which guarantees their long-term stability in electrochemical energy devices. In this review, we have discussed the COFs constructed with different building blocks displaying intrinsic, extrinsic and combined proton conduction under anhydrous conditions. Particularly, we elaborated the role of external proton sources and their interaction with building blocks on the generation and active migration of protons along the pore channels. The choice of proton sources and their effective binding to keep them intact in nanopores of COFs to ensure immobilization of proton sources and thereby achieved the flow of proton. In addition, we have tabulated the recently developed COFs exhibiting anhydrous proton conduction in detail (Table 1). The enhanced stability of some COFs made possible through building blocks that could promote intra and interlayer interactions. For the practical applications, in fact, COFs still have some challenges to be resolved. (i) Till date, very few membrane electrode assemblies (MEAs) were fabricated by using COFs, especially anhydrous proton conducting COFs.^29,43,51,70–72^ The suitable engineering methods need to be developed to validate the potentiality of these COFs in MEAs. (ii) The predominantly employed hydrothermal synthetic route for COFs involving large amount of hazardous organic solvents must be replaced with environmentally benign green synthesis methods. (iii) The stability of COFs may be challenged by external proton sources doped into COFs owing to its leaking tendency over a period of time. Thus, intrinsic proton conducting COFs exhibiting high proton conduction under anhydrous conditions are necessarily to be developed by molecular engineering of tailorable building blocks. The long term of stability of COFs exhibiting steady proton conductivity over a period of time is still need to be extensively investigated before implementing them in fuel cell devices. In future, alternative synthetic approaches (interfacial polymerization, mechanochemical, microwave synthesis, *etc.*) may dominate towards scalable synthesis of COFs from gram scale to kilogram scale for the industrial fabrication of real time devices. (iv) In fact, fuel crossover is an unavoidable phenomenon in fuel cells particularly at the initial stage of operation which pose a serious threat to the operation and stability of proton exchange membranes. Further, acid-leaking also deteriorates the PEM and potentially degrade the fuel cell system. So, the retention of acid within the pores of COFs needs to be achieved to overcome the loss of proton carriers in PEM and to retain the operational stability of fuel cells at high temperature.^73–77^ It is apparent that COFs are potential candidates exhibiting many valuable qualities, and if the above mentioned constrains addressed, COFs exhibiting proton conduction under anhydrous conditions would be an unavoidable platform for proton exchange membrane in electrochemical energy harvesting systems.

**Table 1:** Comparison of COFs exhibiting proton conduction under anhydrous condition

Compound	Working temperature (°C)	Proton conductivity (S cm^−1^)	Stability		Sample measurement type	Testing method	*E*_a_ (eV)	BET surface area (m^2^ g^−1^)	Ref.
			Thermal (°C)	Chemical					
Aza-COF-1	50	10^−8^ to 10^−9^	250	12 M HCl, 14 M KOH	Pellet	Two electrode	0.78	99.0	49
PA@aza-COF-1	—	—	189	—	—		—	5.2	
Aza-COF-2	50	10^−8^ to 10^−9^	250	12 M HCl, 14 M KOH	Pellet		0.96	102.2	
PA@aza-COF-2	—	—	198	—	—		—	10.9	
Tp-azo	—	—	350	HCl (9 N), H_3_PO_4_ (1.5 M), NaOH	—	Quasi four probe	—	1328	50
H_3_PO_4_@Tp-azo	67	6.7 × 10^−5^	—	—	Pellet		0.11	—	
Tp-Stb	59	—	350	HCl (9 N), H_3_PO_4_ (1.5 M), NaOH	—		—	422	
TpBpy-ST	—	—	350	9 N HCl, 3 M H_3_PO_4_, 3 M NaOH	—	Quasi four probe	—	1746	51
PA@TpBpy-ST	100	1.98 × 10^−3^	—	—	Pellet		0.12	—	
TpBpy-MC	—	—	350	9 N HCl, 3 M H_3_PO_4_, 3 M NaOH	—		—	293	
PA@TpBpy-MC	50	1.4 × 10^−2^	—	—	Pellet		0.11	—	
COF-F6	—	—	300	H_3_PO_4_, HCl, HNO_3_	—	Two stainless steel electrode	—	—	52
PA@COF-F6	140	4.2 × 10^−2^	—	—	Pellet		0.09	—	
COF-F8	—	—	300	12 M HCl, 14 M KOH	′′		—	—	
COF-F10	—	—	300	′′	′′		—	—	
TPB-DMeTP-COF	160	9.6 × 10^−11^	440	H_3_PO_4_, THF, CH_3_CN, HCl, NaOH	Pellet	Two steel electrode	—	2894	53
PA@TPB-DMeTP-COF	160	1.91 × 10^−1^	—	—	Pellet		0.34	—	
TPB-DABI-COF	—	—	400	THF, water, HCl (9 M), H_3_PO_4_	—	Two stainless steel electrode	—	865.2	56
H_3_PO_4_@TPB-DABI-COF	160	1.52 × 10^−1^	—	—	Pellet		0.17	—	
TPB-COF	—	—	484	—	—	Two carbon coated Al foil electrode	—	114.4	57
18% H_3_PO_4_@TPB-COF	160	9.95 × 10^−6^	—	—	Pellet		—	—	
TPB-TPT-COF	—	—	504	—	—		—	362.4	
18% H_3_PO_4_@TPB-TPT-COF	160	1.22 × 10^−5^	—	—	Pellet		—	—	
TPT-TPB-COF	—	—	512	—	—		—	836.9	
18% H_3_PO_4_@TPT-TPB-COF	160	1.77 × 10^−5^	—	—	Pellet		—	—	
TPT-COF	—	—	525	H_3_PO_4_, THF, water, acetone, NaOH (14 M), HCl (12 M)	—		—	1909.2	
H_3_PO_4_@TPT-COF	160	1.27 × 10^−2^	—	—	Pellet		0.17	—	
CTF-H	150	3.5 × 10^−7^	650	Water, H_3_PO_4_, NaOH (12 M), HCl (12 M)	′′	Two Ag electrode	—	551	58
H_3_PO_4_@CTF-H	150	1.6 × 10^−1^	—	—	′′		0.25	—	
CTF-L	—	—	650	Water, H_3_PO_4_, NaOH (12 M), HCl (12 M)	—		—	521	
H_3_PO_4_@CTF-L	150	5.1 × 10^−2^	—	—	Pellet		0.26	—	
CPF-1	—	—	>450	—	—	Two Pt electrode	—	582	59
CPF-BT-1	—	—	>450	—	—		—	211.9	
H_3_PO_4_@CPF-1/PVDF	140	9.77 × 10^−3^	—	—	Membrane matrix		0.15	—	
H_3_PO_4_@CPF-BT-1/PVDF	140	1.30 × 10^−2^	—	—	Membrane matrix		0.16	—	
PyTTA-BMTP-COF	—	—	350	HCl (1 M), NaOH (1 M), H_2_O	—	Two electrode	—	368.35	60
PA@PyTTA-BMTP-COF	140	2.6 × 10^−2^	150	—	Pellet		0.22	—	
PyTTA-DHAT-COF	—	—	450	HCl (1 M), NaOH (1 M), H_2_O	—		—	977.03	
PA@PyTTA-DHAT-COF	140	9.2 × 10^−3^	150	—	Pellet		0.078	—	
NKCOF-52	—	—	>250	Acetone, H_3_PO_4_	—	Two carbon paper electrode	—	378	61
H_3_PO_4_@NKCOF-52	160	1.12 × 10^−3^	—	—	Pellet		0.52	—	
NKCOF-53	—	—	>250	Acetone, H_3_PO_4_	—		—	564	
H_3_PO_4_@NKCOF-53	160	1.24 × 10^−2^	—	—	Pellet		0.27	—	
NKCOF-54	—	—	—	Acetone, H_3_PO_4_	—		—	318	
H_3_PO_4_@NKCOF-54	160	2.33 × 10^−2^	>250	—	Pellet		0.29	—	
Tp-Pa-SO_3_H	120	1.7 × 10^−5^	250	—	′′	Quasi four probe	—	1199	62
Phytic@Tp-Pa-SO_3_H	120	7.5 × 10^−5^	—	—	′′		—	—	
Tp-Pa-Py	—	—	250	—	—		—	1534	
phytic@Tp-Pa-Py	120	3.0 × 10^−4^	—	—	Pellet		0.10	—	
Tp-Pa-(SO_3_H-Py)	—	—	250	—	—		—	1235	
Phytic@Tp-Pa-(SO_3_H-Py)	120	5.0 × 10^−4^	—	—	Pellet		0.16	—	
COF-F4	—	—	>300	—	—	Two stainless steel electrode	—	441	63
COF-F6	140	3.63 × 10^−11^	>300	—	Pellet			224	
F6-[dema]HSO_4_-1.5	140	1.33 × 10^−2^	—	—	′′		0.34	—	
COF-C6	—	—	>300	—	—		—	531	
C6-[dema]HSO_4_-1.0	140	2.84 × 10^−3^	—	—	Pellet		0.38	—	
C6-[dema]H_2_PO_4_-1.0	140	3.27 × 10^−5^	—	—	′′		0.59	—	
TB-COF	120	1.52 × 10^−4^	248	—	′′	Two stainless steel electrode	0.37	1077	64
PIL-TB-COF	120	2.21 × 10^−3^	221	—	′′		0.30	23.6	
Py-BT-COF	—	—	400	DMF, MeOH, water, HCl (1 M), NaOH (1 M)	—	Two stainless steel electrode	—	1895	65
Im@Py-BT-COF	130	2.92 × 10^−3^	—	—	Pellet		0.31	—	
Im@Py-TT-COF	130	3.08 × 10^−3^	—	—	′′		0.31	—	
Im@Py-BD-COF	130	8.20 × 10^−4^	—	—	Pellet		0.32	—	
TAPB-DMTP-COF	—	—	—	—	—	Two stainless steel electrode	—	1684	66
trz@TAPB-DMTP-COF	130	3.10 × 10^−3^	—	—	Pellet		0.38	20	
trz@TAPB-DMTP-COF_0.1-BBA_	130	5.05 × 10^−3^	—	—	′′		0.21	1890	
TAP-COF	—	—	—	—	—	—	—	75.0	67
PA@TAP-COF	140	2.65 × 10^−3^	—	—	Pellet		0.46	13.0	
tra@TAP-COF	150	3.82 × 10^−3^	—	—	′′		—	—	
DAAQ-COF	—	—	—	—	—		—	1163.4	
PA@DDAQ-COF	140	9.20 × 10^−3^	—	—	Pellet		0.22	4.0	
PD-COF	—	—	—	—	—		—	519.0	
PA@PD-COF	140	5.51 × 10^−3^	—	—	Pellet		0.24	0.75	
EB-COF	—	—	—	—	—		—	670.3	
PA@EB-COF	180	2.77 × 10^−2^	—	—	Pellet		0.35	6.9	
tra@EB-COF	160	3.25 × 10^−3^	—	—	′′		0.18		
TPB-DMTP-COF	—	<10^−12^	400	DMF, DMSO, THF, MeOH, H_2_O, 12 M HCl, 14 M NaOH	Pellet	Two silver probe	—	2072	68
trz@TPB-DMTP-COF	130	1.1 × 10^−3^	210	—	Pellet		0.21	0.4	
im@TPB-DMTP-COF	130	4.37 × 10^−3^	220	—	Pellet		0.38	2.3	
XJCOF-1	—	—	>260	Water, HCl (3 M)	—	—	—	467	69
XJCOF-2	—	—	>260	′′	—		—	961	
XJCOF-3	—	—	>260	′′	—		—	503	
trz@XJCOF-1	150	4.3 × 10^−3^	>220	—	Pellet		0.25	—	
trz@XJCOF-2	160	1.29 × 10^−2^	>220	—	′′		0.27	—	
trz@XJCOF-3	160	5.6 × 10^−3^	>200	—	′′		0.28	—	
im@XJCOF-1	140	4.38 × 10^−2^	—	—	′′		0.21	—	
im@XJCOF-2	140	3.33 × 10^−2^	—	—	′′		0.24	—	
im@XJCOF-3	140	2.43 × 10^−2^	—	—	′′		0.20	—	

## Conflicts of interest

The authors declare no conflict of interest.

## Supplementary Material
